# The Relationship between Nutritional Status and Body Composition with Clinical Parameters, Tumor Stage, CA19-9, CEA Levels in Patients with Pancreatic and Periampullary Tumors

**DOI:** 10.3390/curroncol28060406

**Published:** 2021-11-17

**Authors:** Aneta Jachnis, Maciej Tomasz Słodkowski

**Affiliations:** Department of General, Gastroenterology and Oncologic Surgery, Medical University of Warsaw, 02-097 Warsaw, Poland; maciej.slodkowski@wum.edu.pl

**Keywords:** nutritional status, pancreatic cancer, body composition, malnutrition

## Abstract

Recent studies have obtained inadequate data on the association between nutritional status, body composition, clinical parameters and tumor stage in patients withpancreatic and periampullary tumors. The purpose of this study was to assess the relationship between nutritional status (NS), body composition (BC) and selected clinical parameters in patients with pancreatic and periampullary cancer, as well as describe the differences between resection and non-resection groups. This is a prospective study of 76 patients with pancreatic and periampullary tumors. We evaluated NS, BMI, body mass loss (BML) and albumin, total protein, CRP, CEA, CA19-9, lipase, amylase, tumor stage, and BC using bioelectrical impedance (BIA). All subjects were divided into resection (*n* = 59) and non-resection (*n* = 17) groups. The non-resection group had a worse NS, as well as increased amylase and WBC, compared to the resection. The selected parameters of BC corresponded to BML albumin, TP, NS, age, BMI, Karnofsky, RBC, HCT and HGB. No associations were found between BC with tumor size, CRP, CA19-9, and CEA. We recorded the relationship between metastasis and NRS, as well as tumor size with SGA. The percentage of BML was positively correlated with age and CRP but negatively correlated with RBC, HGB, HCT and anthropometric measurements. We found many statistical correlations with NS and selected parameters, as well as differences between the resection and non-resection group. The detection of early prognostic factors of nutritional impairments would improve the quality of life and reduce the rate of postoperative complications.

## 1. Introduction

Periampullary carcinoma and pancreatic cancer have generally poor prognoses. Pancreatic cancer (PC) presents a particularly high postoperative complication rate, high mortality, and a low survival rate. The 5-year relative survival in patients with PC is 10.8% (depending on the stage of diagnosis) and increases to 18% in pancreatic tumors and 45% in ampullary carcinoma in the case of a resectable tumor after resection [1,2]. Surgical resection is the only chance for a potential cure. The standard treatment for both pancreatic cancers located at the head of the pancreas and periampullary tumors is pancreatoduodenectomy (PD) and distal or total pancreatectomy for other cancers in the pancreas. Many patients report difficulties eating and gastrointestinal complaints during the perioperative period, which lead to nutritional status impairments [3].

Malnutrition is common in PC patients and is a known risk factor for morbidity and mortality after pancreatic resection. It also worsens other clinical outcomes [4,5]. The literature describes many different methods to assess nutritional status in oncological patients. Not all of them demonstrated sufficient sensitivity in malnutrition screening, particularly in PC patients. In cancer patients, the most commonly used methods of nutritional assessment include Nutritional Risk Score 2002 (NRS-2002), Subjective Global Assessment (SGA). Mini Nutritional Assessment (MNA) and Malnutrition Universal Screening Tool (MUST) [3,6,7,8]. Additionally, the nutritional assessment may contain the body composition analysis and biochemical parameters (total protein, albumin serum level, prealbumin, lymphocyte count among others) [9,10]. In PC, about 80% of patients had elevated carbohydrate antigen (CA19-9), which is the only biomarker in this case accepted by the National Comprehensive Cancer Network (NCCN) in this case [11]. Carcinoembryonic antigen (CEA) is also used in the diagnosis of pancreatic cancer [12]. Both these markers were evaluated in our study and were subjected to a correlation analysis with other parameters.

Moreover, body mass index (BMI) parameters may be used to assess the nutritional status. In PC patients, the mean BMI at the time of diagnosis is 24.8–29 kg/m^2^, which indicates excessive body weight [13,14,15,16,17]. Despite this, approximately 88% of patients with PC are malnourished or have a medium–high risk of malnutrition [5,18]. Current reports have shown that BMI and weight loss were found to be indicators of long-term survival and postoperative outcomes in patients with pancreatic cancer and periampullary malignancy [17,19]. Overweight and obesity increase the incidence of pancreatic fistula, impaired wound healing, and surgical site infection [19,20]. It appears that the key role of this pathogenesis is played by the amount of fat and muscle tissue, but not the body mass itself. Therefore, BMI is not a good indicator of nutritional status when used separately. Traditionally, dual-energy X-ray absorptiometry (DEXA), computer tomography (CT), magnetic resonance imaging (MRI) are the methods that allow for an evaluation of body composition. A bioelectric impedance analysis (BIA) is faster and non-invasive [9]. Among its advantages, one may list its portability and feasibility to be conducted at the patient’s bedside, with the results being available immediately. It is a well-known and widely used tool to assess inter alia fat-free mass and fat tissue for various types of cancer, including pancreatic cancer [21,22]. Several studies have shown the BIA as a helpful measurement assessing nutritional status in a cancer patient, PC, and older subjects [9,23]. The study included both periampullary and pancreatic tumors. In the literature, we encountered examples of both types of tumors included in the study. Pancreatic tumors and periampullary tumors manifest in a similar way, and sometimes it is difficult to differentiate them solely on the basis of imaging techniques. Moreover, the same surgical treatment method (pancreatoduodenectomy) is used in both cases. Our intention was to maximize the generalizability and increase the application of our study to a broader patient population. By including periampullary tumor in the group under study, together with pancreatic cancer, we wanted to additionally assess whether, in this case, the location of the tumor influences the occurrence of differences in nutritional status and compared parameters. However, based on our knowledge, the study described in this paper is one of the first which compares the parameters of nutritional status, body composition, and clinical parameters, tumor stage between resection and non-resection pancreatic and periampullary tumors. We have not found sufficient quantitative data on the above-mentioned parameters in patients with periampullary and pancreatic tumors that qualified for surgery treatment. Therefore, this study was scheduled to investigate appropriate data on nutritional status, body composition, and selected clinical parameters in patients with pancreatic and periampullary malignancies. The primary objective was to determine the relationship between the above-mentioned parameters. The secondary objective was to indicate the difference between resection and non-resection groups.

## 2. Materials and Methods

### 2.1. Study Design

This was a prospective study that collected clinical and nutritional status parameters in patients with pancreatic and periampullary tumors. All subjects were primarily categorized as surgical patients. In sixty-one cases, we assessed the body composition by bioelectrical impedance (BIA). The protocol was approved by the Ethics Board at the Medical University of Warsaw.

### 2.2. Patients Characteristics

A group of ninety-five patients primarily qualified for pancreatic due to malignancies. Nineteen patients were excluded due to histopathological confirmation of severe comorbidities. Accordingly, a total of seventy-six (*n* = 76) patients were included for a short-term outcome analysis. Information regarding nutritional status, anthropometric measurements, bioelectrical impedance, and clinical parameters was collected 24–48 h before the surgery. The patients were admitted to the Department of General, Gastroenterological and Oncological Surgery, at the Medical University of Warsaw in Poland. The inclusion criteria were: pancreatic or periampullary tumor, age > 18 years, absence of other oncological tumors and other oncological therapy in the last 5 years, serious chronic diseases, corticosteroids therapy, no major surgery in the last year.

Imaging scans from computer tomography (CT) or magnetic resonance imaging (MRI) were used for a primary diagnosis. The final recognition was confirmed with histopathology after laparotomy.

Patients’ tumor disease status was classified as adenocarcinoma, adenoplanoepitheliale, acinocellulare, carcinoma papillae vateri, neuroendocrine tumors. To assess the tumor stage, we used the American Joint Committee on Cancer (AJCC) tumor/node/metastasis (TNM) classification. The resection group included patients whose tumor was excised by means of pancreatoduodenectomy or distal pancreatectomy. The non-resection group had only a laparotomy, with biopsy or a palliative procedure performed without pancreatic resection. The resection was not undertaken because there were metastatic tumors in all cases. A single liver metastasis was not considered a contraindication to surgical resection. In our institution, we preoperatively used the generally accepted respectability criteria for pancreatic adenocarcinoma according to the NCCN guidelines [11]. The performance status was objectified by the Karnofsky score. All patients with obstructive jaundice underwent preoperative percutaneous endoscopic bile drainage.

### 2.3. Nutritional Status and Anthropometric Measurements

Nutritional assessment was performed by a registered dietitian including NRS-2002 and SGA. The procedure was conducted within two days before the surgery. We collected data on actual height and body mass, weight loss, pre-diagnosis and actual BMI, the circumference of the arm, calf, and skin fat fold.

Height (cm) and body mass (kg) were measured in the morning, after overnight fasting with barefoot and underwear. Unintentional weight loss was assessed based on usual and actual weight, and usual BMI was calculated based on usual weight before the date of diagnosis.

#### 2.3.1. Body Composition

Body composition parameters were determined by the bioelectrical impedance and included Fat Mass (FM), Fat-Free Mass (FFM), Body Cell Mass (BCM), Phase Angle (PhA), Total Body Water (TBW), extracellular water (ECW), intracellular water (ICW), impedance, and muscle mass, and were assessed by bioelectrical impedance BioScan 920 Maltron. We placed two electrodes on the non-dominant hand and two electrodes on the foot. Finally, the measurement was conducted in 61 (80.3%) patients due to a lack of permission or contraindications to the analysis. We checked statistical differences in body composition parameters, taking gender into account.

#### 2.3.2. Clinical Parameters

Blood samples were collected preoperatively, as part of the clinical routine within 48 h before surgery and included C-reactive protein (CRP), glucose, white blood cell (WBC), hemoglobin (Hb), hematocrit (HCT), red blood cell (RBC), serum albumin level (alb), and total protein (TP), lipase, amylase, alanine aminotransferase (ALT), aspartate aminotransferase (AST). We took the blood samples (5 mL) from a peripheral vein. The CAE, CA19-9 level was evaluated before admission, due to the surgery. Clinicians evaluated the metabolic demands and physical examination.

### 2.4. Statistical Analysis

To compare parameters between the sexes, we used Student’s *t*-test, and between resection and non-resection groups, we performed either Student’s *t*-test or Mann–Whitney’s U test for numerical variables and χ2 for nominal variables (with Fisher’s or Fisher-Freeman–Halton exact tests, where the assumptions for χ2 were violated). The correlation between pairs of variables was assessed by either Pearson’s r test or Spearman’s rho. The statistically significant value was *p* < 0.05.

## 3. Results

### 3.1. Patient’s Characteristics and Differences between Resection and Non-Resection Groups

We evaluated 76 patients with pancreatic cancer and periampullary tumors, i.e., 46.1% women and 53.9% men. After histopathological and intraoperative diagnosis, all subjects were divided into resection *n* = 59 (77.6%) and non-resection *n* = 17 (22.4%) tumor groups. The most frequent tumor was located at the head of the pancreas 72.4% (*n* = 55) and the histopathology type was adenocarcinoma 92.1% (*n* = 70). There were 14.3% (*n* = 11) patients with tumors in the papilla of Vater. The differences in clinicopathological and nutritional assessment characteristics between resection and non-resection tumor groups are described in Table 1.

### 3.2. Nutritional Status

**Figure 1:**
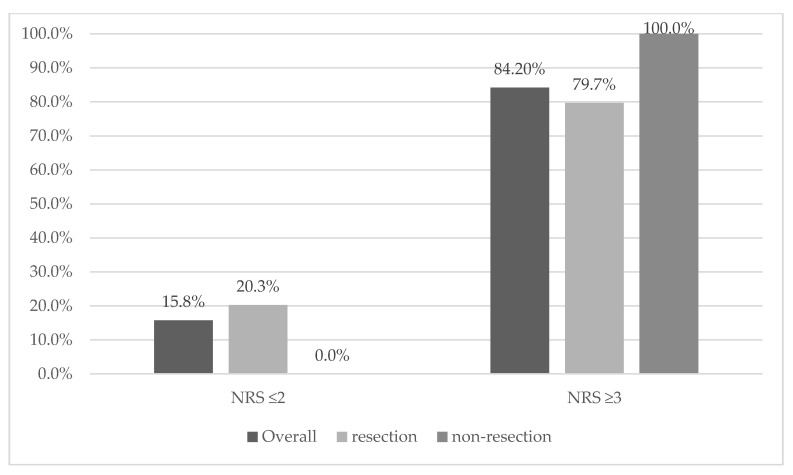
Distribution of the risk of malnutrition based on NRS 2002 compared with overall, resection and non-resection tumor group: NRS ≤ 2: no risk of malnutrition, NRS ≥ 3: risk of malnutrition.

**Figure 2:**
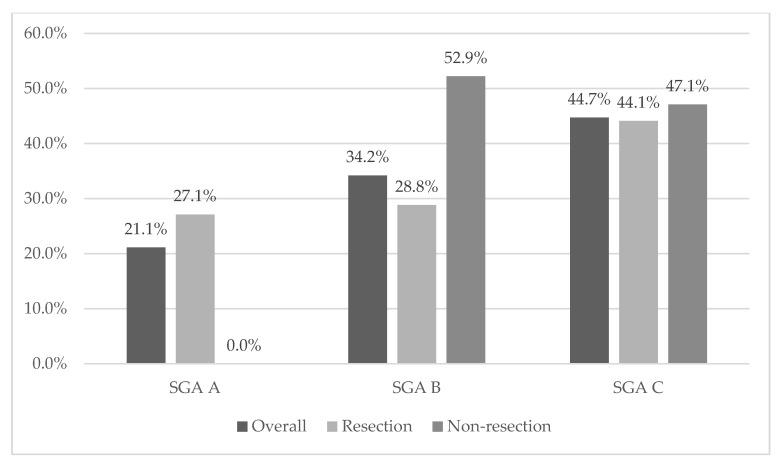
Distribution of the severity of malnutrition based on SGA compared with overall, resection and non-resection: SGA A: well nourished, SGA B: moderately malnourished or at risk of malnutrition, SGA C: severely malnourished.

Malnutrition was recorded in 84.2% (*n* = 64) and 78.9% (*n* = 60) using NRS 2002 and SGA. Severe malnutrition (grade C) was detected in 44.7% (*n* = 34) of overall cases. We recorded the differences in nutritional status compared with resection and non-resection tumor groups (Figure 1 and Figure 2). In the SGA case, all patients in the non-resection group had malnutrition in the case of NRS; patients in the non-resection group were disproportionately more likely to score 6, which indicates severe malnutrition. In terms of other parameters, the analysis showed no statistically significant differences or relationships; see Table 1 and Table 2.

In addition to the above results, the analysis showed statistically significant differences only in the mean level of amylase (*p* = 0.046) and white blood cells WBC (*p* = 0.007)—both parameters were greater in the resection group and red blood cells RBC, which were higher in the non-resection group (*p* = 0.032).

### 3.3. Body Composition

We recorded no differences in body composition between non-resection and resection tumor patients; the mean parameter values are presented in Table 2.

**Table 2:** Comparison of body composition parameters in resection and non-resection tumor groups.

Parameter	Overall *n* = 61 Mean (SD)		Resection *n* = 59 Mean (SD)		Non-Resection *n* = 17 Mean (SD)		*p*
FFM [kg]	48.61	11.04	49.06	11.61	46.80	8.56	0.530
FFM [%]	68.16	10.42	67.74	9.42	69.85	14.20	0.535
FAT [kg]	23.64	10.26	24.06	9.52	21.94	13.20	0.608
FAT [%]	31.85	10.22	32.33	9.27	29.92	13.74	0.468
50 Hz PhA	7.64	1.52	7.67	1.49	7.55	1.68	0.809
BCM [kg]	26.23	5.95	26.45	6.26	25.33	4.62	0.565
TBW [L]	35.03	8.87	35.05	8.92	34.94	9.05	0.970
ICW [kg]	54.59	2.83	54.40	2.87	55.35	2.66	0.303
ECW [kg]	45.40	2.83	45.59	2.87	44.64	2.66	0.302
ICW/ECW *	0.83	0.10	0.84	0.10	0.80	0.09	0.062
Muscle [kg]	22.54	6.59	22.58	6.98	22.39	4.97	0.929
Impedance 50 Hz	541.79	93.95	537.65	96.52	558.67	84.31	0.492

* for ICW/ECW non-parametric U Mann—Whitney’s test was performed. Abbreviations: FFM [kg]—fat free mass in kg, FFM [%]—fat-free mass in %, FAT [kg]—fat mass in kg, FAT [%]—percent of fat mass, 50 Hz PhA—phase angle using frequency 50 Hz, BCM—body cell mass, TBW—total body water, ICW—intra-cell water, ECW–extra cell water.

### 3.4. Relationship between Nutritional Status, Body Composition, and Clinical Parameters

The analysis showed a series of correlations between nutritional status parameters and age, status performance, tumor stage, and other clinical parameters. We found a positive correlation of body mass loss% with icw, 50 Hz impedance, CRP, and age, as well as a significant negative correlation with fat_kg, tbw_lt, ecw%, ecw/icw, arm, calf, BMI, Karnofsky score, RBC, HGB, and HCT. However, in the case of weight loss in kilograms, there was only a significant positive correlation with age, CRP, and BMI_usual, as well as negative correlations with Karnofsky, RBC and HCT scores. Moreover, in the tested sample, there were positive correlations between the NRS score and 50 Hz impedance, tnm_m, CEA, CRP and age, as well as negative correlations between NRS and ecw/icw, arm, calf, Karnofsky score, RBC, HCT and HGB. Positive correlations were also found between alb and fat_kg, arm, BMI, Karnofsky score, RBC, HGB, HCT, as well as a negative correlation with CRP. There was also a positive correlation between tp level and fat_kg, Karnofsky score, RBC, HGB and HCT. SGA was significantly correlated with 50 Hz impedance, CA19-9, CRP, and age (positive correlations), as well as fat_kg, bcm_kg, ecw/icw, arm, calf, fold_tric, BMI, Karnofsky, and HGB scores. Moreover, statistically significantly higher levels of SGA were observed in the group with tumor nodus metastases.

Additionally, we assessed whether the tested parameters differed depending on the presence of cachexia and the risk of malnutrition (NRS score 3 or higher). The group with cachexia had higher CRP and lower fat_kg and Karnofsky scores. However, in the group with NRS, indicating the risk of malnutrition, the levels of icw and CA19-9 were higher, whereas the levels of fat_kg, ecw, ecw/icw, Karnofski’s scale, and RBC were lower. Specific values and significance levels of the analysis are presented in Table 3.

### 3.5. Relationship between Body Compositions with Tumor Stage, Karnofsky Performance, and Other Clinical Parameters

The conducted analysis showed the existence of a series of correlations between body composition and other parameters (Table 4.). There was a statistically significant positive correlation between FFM in kilograms and BMI, BMI_usual, Karnofsky score, arm, calf, and HGB, as well as a negative correlation between FFM_kg and age. Negative correlations were also found between FFM% and BMI, BMI_usual, arm, calf, and fold_tric. Moreover, FAT_kg was found to positively correlate with BMI, BMI_usual, Karnofsky score, arm, calf, fold_tric, RBC, HCT, and HGB. There were also positive correlations of FAT% with almost all these variables, except for Karnofsky score, RBC, HCT, and HGB. Phase angle correlated positively with BMI, Karnofsky score, RBC, HCT and HGB, but negatively with age. BCM in kilograms and TBW_lt correlated positively with BMI, BMI_usual, Karnofsky score, arm, calf, and HGB, and negatively with age. In the case of ECW and ECW/ICW, only significant positive correlations between BMI, arm, and calf were observed, and in the case of ICW, these correlations were negative. The analysis also showed a significant positive correlation between muscle_kg and arm, calf, and HGB, and a negative correlation with age. We also observed that the 50 Hz impedance positively correlates with tmn_m and negatively with BMI, BMI_usual, Karnofsky, arm, and calf scores.

## 4. Discussion

### 4.1. Malnutrition, Weight Loss and BMI

In our study, 78–84% of patients were malnourished or had a risk of malnutrition depending on the nutritional assessment method. Cachexia was related to 68.4% of cases. In the resection group, all patients had malnutrition based on SGA and NRS 2002. In Bicackli et al.’s study, malnutrition was recorded in over 85% of patients with PC using PG-SGA [5]. In most studies using NRS 2002, approximately 52–88% of resectable PC patients presented moderate to severe risk of malnutrition, which is consistent with our observations [18]. Nutritional Risk Score 2002 was found to be a good factor determining malnutrition and outcomes after surgery in PC [24]. However, in some analyses, NRS 2002 was not sufficiently sensitive to detect malnutrition or cachexia [25]. Therefore, in our analyses, we used both NRS with SGA and we assessed parameters of impaired nutritional status, mainly unintentional weight loss. In the early stage of pancreatic cancer, almost 50% of patients undergoing pancreaticoduodenectomy presented a loss of body mass [25]. In pancreatic ductal adenocarcinoma PDAC, loss of weight occurs in 88.6% of cases, with a median of 6.8 kg [14]. We have reported similar results for body mass loss in 89.5% of patients, and the mean value was 8.26 kg (9.98%). An increased percent of body mass loss was associated with a decreased ECW%, TBW Lt, FAT kg but increased ICW%. There were no correlations between muscle mass and weight loss. Similarly, in Morgado et al.’s study, no statistically significant association was found in the percent of weight loss with skeletal muscle (kg) and percent of body fat [26]. Our result suggests that weight loss was mainly related to body fat and total water, which is consistent with the data from observations in other authors’ research [21,22]. Another common parameter used in nutritional status assessment is body mass index (BMI). Based on the current knowledge, the mean BMI value in PC patients is 22.9–29.2 kg/m^2^, and this is dependent on the disease stage [3,13,14,15,16,17,27]. We found a positive correlation between usual BMI and weight loss (kg) during the time of the diagnosis. In other studies, the higher usual body mass and pre-diagnostic BMI was associated with greater loss of weight and more advanced stage of pancreatic cancer [14,24,27]. The mean BMI and primarily resectable surgical recognition may erroneously indicate a relatively good nutritional status. However, when assessing the mean values of body weight loss at 9–12% and 7–10 kg, depending on the group, it can be seen that the majority of patients qualifying for surgical treatment are malnourished. The mean percent of FAT in these results indicates an excessive amount of fat relative to population norms. Therefore, even BMI correlates with parameters of nutritional status, and weight loss is an insufficiently sensitive predictor of malnutrition in PC patients, particularly for sarcopenia assessment, but can be used as an auxiliary tool. BMI can provide incomplete data, especially for body composition, fat distribution and fat-free mass resources. Nevertheless, it could be a good indicator of postoperative outcome and overall survival (OS).

### 4.2. Age

The mean age of patients in our study was 65 years. Generally, PC patients are elderly, and the median age of PC diagnosis is over 70 years. The risk of developing PC increases with age [28]. It is estimated that only one-fifth of patients who develop pancreatic cancer are under 60 [29]. We observed a negative correlation between age and body mass and a positive one with percent of weight loss. Older age had an impact on the body composition in our patients, with decreased fat-free mass [kg], TBW [lt], phase angle, BCM [kg] and muscle mass [kg]. Advanced age was associated with lower albumin and increased CRP (the result was close to statistical significance *p* = 0.064). Geriatric patients have an increased prevalence of frailty and often have poor outcomes. Furthermore, there is a greater likelihood of developing postoperative complications and increased risk of mortality [29].

### 4.3. Albumin and Protein

Patients with PC in the pre-operative period presented a mean albumin level 3.7–4.0 g/dL and 6.8 g/dL total protein level depending on the study [3,30,31,32]. In Ferrucci et al.’s study of locally advanced pancreatic cancer, the mean serum albumin level was 3.7 g/dL [16]. We observed relatively high levels of albumin and total protein in subjects 3.74 g/dL and 6.52 g/dL. Despite this, there was a negative correlation between albumin with percent of body weight loss and a positive one with BMI and fat tissue in kg. In contrast to our results, in the study of patients with resectable PDAC, no correlation was found between BMI and albumin [20]. Low albumin level was described as a good indicator to predict outcomes in pancreatic cancer and intraoperative complications in pancreatic surgery, which is an important parameter when assessing the general clinical condition of the patients [20,30,33].

### 4.4. CRP

C-reactive protein could be increased due to an inflammation as well as trauma or advanced cancer disease, and may be associated with poor outcomes and increased postoperative complications [33]. The CRP value > 10 g/dL had a statistical impact on OS in PC, as well as in advanced PC and APD receiving chemotherapy. The value of CRP was confirmed to be an independent prognostic factor for OS by multivariate analysis [33,34,35]. Higher CRP values correlate with an increased risk of malnutrition in ours and other studies and is increased in patients with severe malnutrition [36]. We found a correlation between CRP and albumin, age, weight loss, but there was no association with BMI and CRP level. Moreover, no statistically crucial differences were observed in non-resection or resection groups, and there were no correlations between body composition parameters, except for a positive correlation with FAT_kg. We were unable to find other studies evaluating these parameters in the selected group.

### 4.5. CA19-9 and CEA

In our subjects the preoperative mean level of CA19-9 was 548.27 U/mL and CEA 6.46 µg/L. All the patients with obstructive jaundice underwent preoperative percutaneous or endoscopic bile drainage. Therefore, it is unlikely that the difference in the CA19-9 levels was due to obstruction of the bile duct. In the advanced stage of PC, high CA19-9 and CEA levels were significantly associated with decreased OS [34,35,37]. We recorded that CA19-9 only had a negative association with malnutrition. CEA correlated with muscle (kg), either phase angle, FFM kg, but this was not statistical. Patients with a high risk of malnutrition had a higher level of CA19-9, and NRS was positively correlated with CEA. Patients in the resection group did not have a significantly higher level of CEA than the ones belonging to the group with the non-resection (*p* = 0.068). Regarding the values of tumor markers CEA and CA19-9, whose values we compared in the resection and non-resection group, there were no statistically significant differences. The lack of statistical significance in the CEA and CA19-9 levels between the resection and non-resection groups is difficult to explain. To some extent, this could be attributed to different proportions of patients with the papilla of Vater and pancreatic cancer. Additionally, although all the patients with obstructive jaundice underwent preoperative percutaneous or endoscopic bile drainage, persistent hyperbilirubinemia in selected patients might have affected the CA19-9 levels. We did not find any relationship between BMI or usual BMI and CEA or CA19-9 level.

### 4.6. BIA Parameters

To date, the correlation between body composition assessed with BIA and other parameters in PC patients has not been investigated and described thoroughly enough. The most important parameters of body composition include fat tissue (FM), fat-free mass (FFM) and body cell mass (BCM). Cai et al.’s study of patients with chronic radiation enteritis indicated a significant correlation between BCM and FM [kg] with CRP and albumin [38]. In the study of patients with operable pancreatic adenocarcinoma, the body composition parameters were not a good indicator to predict postoperative outcomes. There was a recorded decrease in the percent of FM in pancreatic cancer patients vs. the control group, but only in the female group [30]. However, BIA may be used as a tool for estimating muscle mass, particularly after applying appropriate cut-off points and the specificity of the studied population [39]. Muscle mass is an important parameter, which is used for diagnosing sarcopenia. It correlates with postoperative outcomes and survival time after pancreatic surgery [40,41,42]. The most common parameter of BIA performed in oncology is a phase angle (PhA) [10,23]. In studies of cancer patients, PhA strongly correlates with the degree of malnutrition, and a lower phase angle is associated with lower survival [10,23,43]. In patients with mild or low risk of malnutrition, PhA is not a sensitive tool in nutritional assessment [44]. However, in advanced pancreatic cancer, PhA is a prognostic factor [45]. Referring to our results in patients with cachexia, we recorded no differences in BIA parameters compared to non-cachectic ones, except FAT kg. Patients at risk of malnutrition had a lower value of fat mass in kilogram and ECW%, ECW/ICW, but a higher ICW% compared to patients, with no risk of malnutrition. No body composition factor corresponded to the degree of malnutrition. Most body composition parameters correlated with age, BMI, usual BMI, and loss of body mass. The present study revealed that the phase angle was positively correlated with Karnofsky score, BMI, hemoglobin, and hematocrit values. This indicates the significance of these parameters in PC and periampullary tumor patients. In Tumas et al.’s study, decreased PhA was recorded in 39% and lower fat-free mass index (FFMI) in 3.4% of early PC patients compared to the normal range [25]. FFM may be correlated with albumin level in patients with cirrhosis, but it should be recorded and assessed for each individual, like PhA. The PhA value is dependent on the stage of disease and the type of cancer [23]. The most recommended way of using BIA parameters is to follow the subjects throughout treatment and refer the results to individual observations [23]. According to our experience, none of the body composition parameters differed between the resection and non-resection groups. However, we noted significant differences between the sexes (*p* < 0.001), but they did not concern the phase angle and FAT [kg]. In the future, analyses should consider the above-mentioned observations. Additionally, we observed more correlations between the phase angle and other parameters when we used 5 Hz instead 50 Hz (these data are not included in this analysis).

### 4.7. TNM

Our analysis indicates that patients with distant metastasis had a lower BMI than patients without metastasis. We did not find significant differences in other parameters of TNM and BMI. An increased risk of malnutrition was associated with higher metastasis incidences. Another study shows that PC patients with increased usual BMI presented metastasis more often than those with normal BMI [27]. In the analyses of the colorectal cancer patients, McSorley et al. did not find any relationship between TNM stage and body composition measured by computer tomography CT [46]. This result corresponds to ours, where we only found a positive correlation with TNM_M and impedance, but there were no differences in percent of body weight loss, albumin, total protein values, and degree of malnutrition according to TNM.

Comparing the data from various publications, one should consider the differences between determined parameters. In our work, we noted the most discrepancies in the results in the case of weight loss (the percentage vs. kilograms of body weight), with different correlations. Significant differences also occurred when determining the relationship between the parameters FFM% vs. FFM kg and FAT% vs. FAT kg. The above parameters were interchangeably evaluated depending on the publication. This may lead to errors in the interpretation of several different works.

### 4.8. Limitations

The limitations of this study included a small sample as well as relatively insufficient malnutrition indicators, especially lymphocytes or prealbumin. Due to the high median age in the study, a more valid screening tool is recommended, i.e., the Malnutritional Universal Screening Tool (MUST), especially for elderly patients [47]. Future analyses should include a post-operative analysis of the nutritional and metabolic status and relate to a larger group of subjects.

## 5. Conclusions

This study has shown that patients with resection pancreatic and periampullary tumors have a generally poor nutritional status and significant weight loss, but relatively high BMI and high albumin and total protein levels.

The body composition of patients with pancreatic and periampullary cancer does not significantly differ between resection and non-resection patients. Additionally, the degree of malnutrition in patients with early-stage cancer does not affect the differences in body composition. However, less severe malnutrition is more common in resection, and severe malnutrition is more common in non-resection. A proper, detailed assessment of nutritional status in patients qualified for pancreatic surgery, including body composition measurements, is particularly important, and should be an indispensable element of the individual nutritional therapy. To obtain correct body composition results, gender differences should be considered. Future investigation, referring to body composition parameters, could indicate a strong prognostic value of fluid and fat distribution in patients with pancreatic cancer. Also theearly indicators of the postoperative complications and long-term outcomes in this group of cancers shouldof interest to future research.

Due to the poor prognosis and an increased mortality rate in patients undergoing pancreatic surgery for cancer, it is necessary to apply an individualized diet therapy, including clinical nutrition. This step could improve nutritional status and clinical parameters and decrease the risk of intraoperative outcomes, with a reduction in the adverse effects of cancer treatment.

## Figures and Tables

**Table 1:** Clinicopathological and nutritional assessment characteristics between resection and non-resection tumor groups.

Parameter	*p*	Overall		Resection		Non-Resection	
		*n* = 76		*n* = 59		*n* = 17	
Age at diagnosis, mean (SD) [year]	0.313	65.32 (9.65)		64.71 (9.18)		67.41 (11.17)	
Gender							
M *n* (%)	0.312	41 53.9%		30	50.80%	11	64.70%
F *n* (%)		35 46.1%		29	49.20%	6	35.30%
BMI at diagnosis, mean (SD) [kg/m^2^]	0.679	25.16 (4.59)		25.28 (4.54)		24.75 (4.85)	
Usual BMI, mean (SD) [kg/m^2^]	0.767	27.97 (4.83)		27.88 (4.80)		28.28 (5.08)	
NRS 2002: [*n*, %] *							
2	**0.024**	12	15.80%	12	20.30%	0	0.00%
3		18	23.70%	15	25.40%	3	17.60%
4		15	19.70%	11	18.60%	4	23.50%
5		23	30.30%	18	30.50%	5	29.40%
6		8	10.50%	3	5.10%	5	29.40%
SGA [*n*, %]	**0.032**						
A		16	21.10%	16	27.10%	0	0.00%
B		26	34.20%	17	28.80%	9	52.90%
C		34	44.70%	26	44.10%	8	47.10%
Weight loss at diagnosis, mean [kg]	0.247		8.26		7.76		10
Weight loss at diagnosis, mean [%]	0.157		9.98	9.27		12.4	
≤5% (*n*) [%]	0.63	24	31.60%	20	33.90%	4	23.50%
>5% (*n*) [%]		19	25.00%	15	25.40%	4	23.50%
>10%(*n*) [%]		33	43.40%	24	40.70%	9	52.90%
Malnutrition risk NRS 2002 (≥3) *n* (%) *	0.058		64 84.2%	47	79.70%	17	100.00%
Malnutrition SGA *n* (%)							
(stage B, C) *n* (%) *	**0.016**		60 78.9%	43	72.90%	17	100%
Diabetes mellites present at diagnosis, *n* (%)	0.231		41 53.9%	34	57.60%	7	41.20%
Serum albumin level [g/dL] mean	0.389		3.74	3.79		3.6	
Total protein level [g/dL] mean	0.767		6.52	6.53		6.47	
Glucose [mg/dL] mean *	0.874		140.36	139.12		144.59	
Lipase [U/L] mean *	0.316		121.36	122.02		119.06	
Amylase [U/L] mean *	**0.046**		71.23	79.55		42.82	
Alt [U/L] mean *	0.097		83.43	86.97		71.18	
Ast [U/L] mean *	0.414		115.24	136.31		42.12	
WBC [G/L] mean *	**0.007**		8.39	9.10		5.96	
HCT [%] mean	0.093		38.13	38.6		36.52	
HGB [g/dL] mean *	0.074		12.95	13.22		12.04	
RBC [T/L] mean *	**0.032**		8.10	6.85		12.38	
CEA mean *	0.068		6.46	4.13		15.6	
CA19-9 mean *	0.526		548.27	621.39		275.28	
C-reactive protein (CRP 1) [mg/dL] mean *	0.205		16.52	16.86		15.33	
Cachexia	0.418		52 (68.4%)	39 (66.1%)		13 (76.5%)	
Tumor size (*n*) [%] *							
T1	0.836	1	1.30%	1	1.70%	0	0.00%
T2		15	19.70%	13	22.00%	2	11.80%
T3		55	72.40%	41	69.50%	14	82.40%
T4		5	6.60%	4	6.80%	1	5.90%

Variables marked with * were analysed with U Mann–Whitney test (for continuous variables) and Fisher’s exact test or Fisher–Freeman–Halton test for nominal variables. Abbreviations: M—males, F—Females, BMI—body mass index, usual BMI—body mass index in pre-diagnosis time, NRS 2002—Nutritional Risk Score 2002, SGA—Subjective Global Assessment, WBC—white blood cells, HCT—hematocrit, RBC—red blood cells. The bold type indicates statistical significance (*p* < 0.05).

**Table 3:** Correlations and differences in age, status performance, tumor stage, and other clinical parameters depending on nutritional status, weight loss, albumin, total protein and cachexia.

Selected Clinical Parameters		b.Mass.%	b.Mass_Loss	NRS	ALB	TP	NRS ≥ 3	SGA	Cachexia
ffm_kg		−0.17	0.04	−0.16	0.01	−0.02	−0.19	−0.20	0.44
	*p*	0.182	0.78	0.228	0.935	0.896	0.847	0.135	0.66
Ffm %		0.24	0.13	0.08	−0.24	−0.21	1.21	0.11	1.55
	*p*	0.062	0.303	0.546	0.069	0.111	0.231	0.41	0.125
fat_kg		**−0.39**	−0.19	−0.20	**0.32**	**0.30**	**−2.37**	**−0.26**	**−2.09**
	*p*	**0.002**	0.146	0.128	**0.014**	**0.026**	**0.021**	**0.049**	**0.041**
fat%		−0.24	−0.12	−0.05	0.25	0.22	−1.12	−0.07	−1.50
	*p*	0.065	0.342	0.683	0.057	0.098	0.268	0.572	0.14
50 hz_pa		−0.22	−0.2	−0.17	0.17	0.05	−0.52	−0.15	−0.63
	*p*	0.083	0.128	0.184	0.207	0.687	0.604	0.258	0.53
bcm_kg		−0.24	−0.02	−0.21	0.09	0.06	−0.9	**−0.26**	−0.12
	*p*	0.07	0.864	0.106	0.486	0.666	0.372	**0.043**	0.908
tbw_lt		**−0.26**	−0.03	−0.15	0.08	0.06	−0.65	−0.22	0.05
	*p*	**0.048**	0.808	0.253	0.532	0.633	0.516	0.095	0.96
Icw %		**0.31**	0.13	0.24	−0.03	0.09	**2.13**	0.24	1.47
	*p*	**0.017**	0.323	0.063	0.83	0.508	**0.037**	0.062	0.147
Ecw %		**−0.31**	−0.13	−0.24	0.03	−0.09	**−2.13**	−0.24	−1.47
	*p*	**0.017**	0.324	0.063	0.833	0.502	**0.037**	0.063	0.148
ecw/icw *		**−0.29**	−0.20	**−0.27**	0.08	0.04	**−2.10 ^b^**	**−0.25**	−1.56
	*p*	**0.024**	0.117	**0.039**	0.536	0.795	**0.036**	**0.049**	0.119
musle_kg		−0.06	0.11	−0.03	0.08	0.11	1.03	−0.08	1.22
	*p*	0.632	0.383	0.806	0.571	0.433	0.308	0.535	0.228
50 hz_imp		**0.30**	0.10	**0.26**	−0.10	0.01	1.43	**0.32**	1.19
	*p*	**0.018**	0.433	**0.044**	0.47	0.972	0.159	**0.012**	0.237
Arm		**−0.46**	−0.25	**−0.38**	**0.28**	0.16	−1.54	**−0.3**	−1.63
	*p*	**<0.001**	0.058	**0.003**	**0.04**	0.255	0.129	**0.022**	0.11
Calf *		**−0.38**	−0.26	**−0.4**	0.13	0.01	−1.31	**−0.33**	−1.38
	*p*	**0.004**	0.053	**0.002**	0.349	0.97	0.189	**0.011**	0.169
fold_tric *		−0.28	−0.20	−0.25	0.19	0	−0.83	**−0.33**	−1.63
	*p*	0.055	0.17	0.081	0.192	1	0.405	**0.022**	0.104
bmi_usual		0.17	**0.37**	0.19	0.13	0.08	1.19	0.11	1.19
	*p*	0.15	**0.001**	0.102	0.274	0.528	0.236	0.33	0.237
BMI		**−0.36**	−0.13	−0.19	**0.32**	0.19	−1.07	**−0.27**	−1.98
	*p*	**0.001**	0.252	0.101	**0.006**	0.115	0.288	**0.018**	0.052
Karnofsky *		**−0.45**	**−0.38**	**−0.52**	**0.41**	**0.27**	**−3.51 ^b^**	**−0.35**	**−2.84 ^b^**
	*p*	**<0.001**	**0.001**	**<0.001**	**<0.001**	**0.024**	**<0.001**	**0.002**	**0.005**
tnm_t		0.16	0.16	0.13	−0.04	0.07	0.64	0.15	0.55
	*p*	0.173	0.168	0.277	0.758	0.576	0.526	0.189	0.586
tnm_*n*		−1.29	−1.45	−1.34	0.31	−0.03	0.73	−1.48	0.03
	*p*	0.202	0.151	0.183	0.756	0.973	0.392	0.142	0.855
tnm_m		0.05	−0.03	**0.24**	−0.1	−0.07	−1.37	0.16	−0.76
	*p*	0.675	0.782	**0.049**	0.416	0.59	0.174	0.183	0.452
CA19−9 *		0.16	0.17	0.15	−0.23	−0.09	**−2.20 ^a^**	**0.26**	−0.53
	*p*	0.181	0.153	0.215	0.061	0.478	**0.028**	**0.026**	0.597
Cea *		0.16	0.21	**0.25**	−0.13	0.01	−0.91	0.13	−0.13
	*p*	0.214	0.097	**0.044**	0.318	0.942	0.364	0.306	0.896
Rbc *		**−0.25**	**−0.23**	**−0.34**	**0.37**	**0.36**	**−1.97 ^b^**	−0.22	−0.94
	*p*	**0.03**	**0.047**	**0.003**	**0.001**	**0.002**	**0.049**	0.06	0.349
Hgb *		**−0.34**	**−0.3**	**−0.38**	**0.53**	**0.46**	−1.64	**−0.31**	−1.67
	*p*	**0.002**	**0.009**	**0.001**	**<0.001**	**<0.001**	0.101	**0.007**	0.095
Hct		**−0.25**	−0.21	**−0.26**	**0.47**	**0.43**	−0.82	−0.22	−0.84
	*p*	**0.034**	0.072	**0.026**	**<0.001**	**<0.001**	0.414	0.057	0.406
Crp *		**0.30**	**0.35**	**0.33**	**−0.26**	−0.11	−1.15	**0.35**	**−2.13 ^a^**
	*p*	**0.009**	**0.002**	**0.004**	**0.028**	0.363	0.25	**0.002**	**0.033**
Age		**0.30**	**0.23**	**0.36**	−0.22	−0.10	−1.95	**0.29**	1.19
	*p*	**0.008**	**0.041**	**0.001**	0.06	0.408	0.055	**0.01**	0.236

Variables marked with * were analyzed with non-parametric tests, and Spearman’s rho was reported instead of Pearson’s r and Z for Mann–Whitney tests instead of *t* statistics for Student *t*-test; for cachexia and NRS ≥ 3 positive t value indicates that a higher mean was observed in the group with cachexia or with NRS equal to or greater than 3. For Mann–Whitney’s test, significant results with higher average rank in the group with cachexia or malnutrition risk was marked with ^a^, and with lower average rank in this group—^b^. Abbreviations: b.mass.%—percent of body mass loss, b.mass_loss—body mass loss in kg, NRS—Nutiritional Risk Score, AlB—albumin, TP—total protein, NRS ≥ 3-Nutritianl Risk Score—risk of malnutrition, SGA—Subjective Global Assessment, arm–arm circumferce, calf–calf circumference, fold_tric—skinfold of triceps, bmi_usual—body mass index in pre-diagnosis time, TNM_T—tumor size, TNM_N—nodus, TNM_M—metastases, FFM [kg]—fat-free mass in kg, FFM [%]—fat-free mass in %, FAT [kg]—fat mass in kg, FAT [%]—percent of fat mass, 50 Hz PhA—phase angle using frequency 50 Hz, BCM—body cell mass, TBW—total body water, ICW—intra-cell water, ECW—extra cell water, WBC—white blood cells, HCT—hematocrit, RBC—red blood cells. The bold type indicates statistical significance (*p* < 0.05).

**Table 4:** Correlations between body composition and other parameters and differences in body composition parameters depending on tumor size.

Selected Clinical Parameters		Ffm_kg	Ffm%	Fat_kg	Fat%	50 hz_pa	Bcm_kg	Tbw_lt	Icw%	Ecw%	Ecw/Icw *	Musle_kg	50 hz_imp
age		**−0.31**	−0.06	−0.07	0.06	**−0.39**	**−0.36**	**−0.31**	0.05	−0.05	−0.03	**−0.25**	0.07
	*p*	**0.015**	0.666	0.599	0.651	**0.002**	**0.004**	**0.017**	0.699	0.697	0.849	**0.05**	0.592
bmi		**0.31**	**−0.74**	**0.93**	**0.76**	**0.32**	**0.36**	**0.44**	**−0.35**	**0.35**	**0.35**	0.19	**−0.52**
	*p*	**0.016**	**<0.001**	**<0.001**	**<0.001**	**0.013**	**0.005**	**<0.001**	**0.007**	**0.007**	**0.006**	0.153	**<0.001**
bmi_usual		**0.26**	**−0.66**	**0.78**	**0.68**	0.23	**0.29**	**0.36**	−0.24	0.24	0.2	0.19	**−0.43**
	*p*	**0.042**	**<0.001**	**<0.001**	**<0.001**	0.078	**0.026**	**0.005**	0.069	0.07	0.131	0.154	**<0.001**
Karnofsky *		**0.26**	−0.23	**0.33**	0.23	**0.57**	**0.28**	**0.33**	−0.09	0.09	0.10	0.18	**−0.33**
	*p*	**0.044**	0.07	**0.01**	0.071	**<0.001**	**0.029**	**0.009**	0.496	0.496	0.454	0.158	**0.008**
arm		**0.53**	**−0.47**	**0.74**	**0.49**	0.18	**0.54**	**0.59**	**−0.38**	**0.38**	**0.41**	**0.39**	**−0.49**
	*p*	**<0.001**	**<0.001**	**<0.001**	**<0.001**	0.17	**<0.001**	**<0.001**	**0.004**	**0.004**	**0.002**	**0.003**	**<0.001**
Calf *		**0.38**	**−0.40**	**0.63**	**0.40**	0.18	**0.39**	**0.44**	**−0.44**	**0.44**	**0.44**	**0.30**	**−0.47**
	*p*	**0.004**	**0.002**	**<0.001**	**0.002**	0.188	**0.003**	**0.001**	**0.001**	**0.001**	**0.001**	**0.026**	**<0.001**
fold_tric *		0.08	**−0.57**	**0.49**	**0.57**	0.07	0.07	0.12	−0.23	0.23	0.23	0	−0.11
	*p*	0.588	**<0.001**	**0.001**	**<0.001**	0.632	0.636	0.434	0.124	0.124	0.117	0.992	0.459
tnm_t		−0.06	−0.10	0.11	0.06	−0.01	−0.04	−0.03	0.20	−0.20	−0.25	0.03	0.09
	*p*	0.671	0.451	0.416	0.643	0.963	0.756	0.822	0.125	0.123	0.051	0.82	0.488
tnm_*n*		0.59	0.79	−0.93	−0.19	0.4	0.35	0.33	−1.16	1.18	−1.51	−0.09	−0.72
	*p*	0.558	0.434	0.359	0.852	0.69	0.724	0.74	0.25	0.244	0.13	0.925	0.472
tnm_m		−0.20	0.10	−0.18	−0.10	−0.09	−0.23	−0.19	0.14	−0.14	−0.20	−0.16	**0.31**
	*p*	0.134	0.466	0.193	0.435	0.498	0.089	0.153	0.311	0.316	0.146	0.245	**0.017**
CA19−9 *		−0.24	−0.22	0.05	0.19	−0.07	−0.23	−0.25	0.08	−0.08	−0.12	−0.22	0.09
	*p*	0.078	0.103	0.719	0.149	0.603	0.083	0.063	0.54	0.54	0.366	0.102	0.487
Cea *		−0.24	−0.07	0.12	0.06	−0.26	−0.22	−0.20	−0.03	0.03	−0.03	−0.25	0.05
	*p*	0.10	0.634	0.409	0.679	0.068	0.117	0.164	0.861	0.861	0.813	0.08	0.725
Crp *		0.17	0.06	−0.01	−0.04	0	0.11	0.17	0.07	−0.07	−0.07	0.19	0.02
	*p*	0.215	0.644	0.965	0.737	0.998	0.424	0.213	0.602	0.602	0.60	0.161	0.902
Rbc *		0.17	−0.18	**0.31**	0.20	**0.26**	0.20	0.16	0.15	−0.15	−0.13	0.15	−0.06
	*p*	0.183	0.159	**0.017**	0.129	**0.04**	0.12	0.209	0.266	0.266	0.308	0.268	0.648
hct		0.14	−0.17	**0.29**	0.2	**0.26**	0.19	0.15	0.14	−0.14	−0.16	0.16	−0.04
	*p*	0.278	0.182	**0.026**	0.12	**0.046**	0.142	0.247	0.275	0.275	0.219	0.226	0.733
Hgb *		**0.31**	−0.14	**0.32**	0.14	**0.29**	**0.36**	**0.30**	0.13	−0.13	−0.12	**0.32**	−0.15
	*p*	**0.018**	0.285	**0.014**	0.274	**0.023**	**0.005**	**0.021**	0.328	0.328	0.356	**0.012**	0.241

Variables marked with * were analysed with non-parametric Spearman’s rho test for correlations and Mann-Whitney’s U for mean comparison. Abbreviations: arm–arm circumferce, calf–calf circumference, fold_tric–skinfold of triceps, bmi_usual–body mass index in pre-diagnosis time, TNM_T–tumor size, TNM_N–nodus, TNM_M–metastases, FFM [kg]–fat free mass in kg, FFM [%]–fat free mass in %, FAT [kg]–fat mass in kg, FAT [%]–percent of fat mass, 50 Hz PhA–phase angle using frequency 50 Hz, BCM–body cell mass, TBW–total body water, ICW–intra cell water, ECW–extra cell water, WBC–white blood cells, HCT–hematocrit, RBC–red blood cells. The bold type indicates statistical significance (*p* < 0.05).

## Data Availability

The data presented in this study are available on request from the corresponding author.
